# Supplementary material for: DNA Aptamers against Vaccinia-Related Kinase (VRK) 1 Block Proliferation in MCF7 Breast Cancer Cells
Source: Pharmaceuticals (Basel). 2021 May 17;14(5):473. doi: 10.3390/ph14050473 (PMC8156982; doi:10.3390/ph14050473)
Supplement: Supplementary file 1 [file pharmaceuticals-14-00473-s001.zip › pharmaceuticals-1176258-supplementary.pdf]

Table S1. Sequences of the aptamers obtained after 6 rounds of SELEX. The selected aptamers are bolded.

	Sequence	nt
**apVRK8**	**ACGCTCGGATGCCACTACAGGGAGGGGGGGGGAAAGTAAGCGGGGGGTCGGCGGCCTCATGGACGTGCTGGTGA**	74
apVRK13	ACGCTCGGATGCCACTACAGCGAGTACGTTGAACTTATCTCATCCCTTCTTTTGCCTCATGGACGTGCTGGTGA	74
apVRK21; 22	ACGCTCGGATGCCACTACAGCCCATGAATGTTTCCTTAGTTACGCATTTCTTCGCCTCATGGACGTGCTGGTGA	74
apVRK27	ACGCTCGGATGCCACTACAGGAGGGCGGGTGGGGGGGGGCTGGCGGTGGTCTGGCCTCATGGACGTGCTGGTGA	74
**apVRK28**	**ACGCTCGGATGCCACTACAGGGACGGCGGATGGGGGCGGTGGGTGGGTTTCTTGCCTCATGGACGTGCTGGTGA**	74
**apVRK33**	**ACGCTCGGATGCCACTACAGGTTGCGAGGTGGGGGGTGGGTAGGGTGGGAAGTGCCCTCATGGACGTGCTGGTGA**	74
apVRK41	ACGCTCGGATGCCACTACAGGGGGCGTGGGATGCCACATTCTTTAAGTTGGGTGCCTCATGGACGTGCTGGTGA	74
apVRK42	ACGCTCGGATGCCACTACAGCCCTAATCTGTCGCTTTGATTTTTTTTACCTCTGCCTCATGGACGTGCTGGTGA	74
apVRK44	ACGCTCGGATGCCACTACAGGGGGCAGGGATGGAAAGTAAGATGTGTGAGGCTGCCTCATGGACGTGCTGGTGA	74

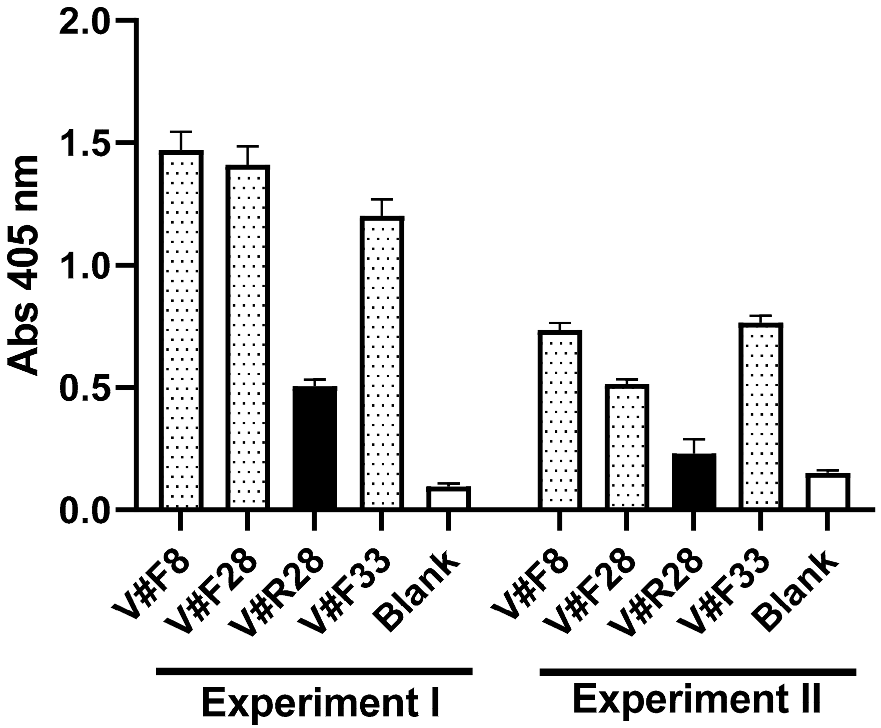

Figure S1: Individual ELONA assays with the four selected aptamers chemically synthesized. Recombinant protein VRK1 (200 ng/well; 4.3 pmol/well) was incubated with digoxigenin-labeled apVRKF8 (V#F8), apVRKF28 (V#F28), apVRKR28 (V#R28) and apVRKF33 (V#33) and ELONA assay performed as described in the Materials and Methods Section using the aptamers at a concentration of 1 ng/μL (40 nM). The results of two independent experiments performed in triplicate are shown.

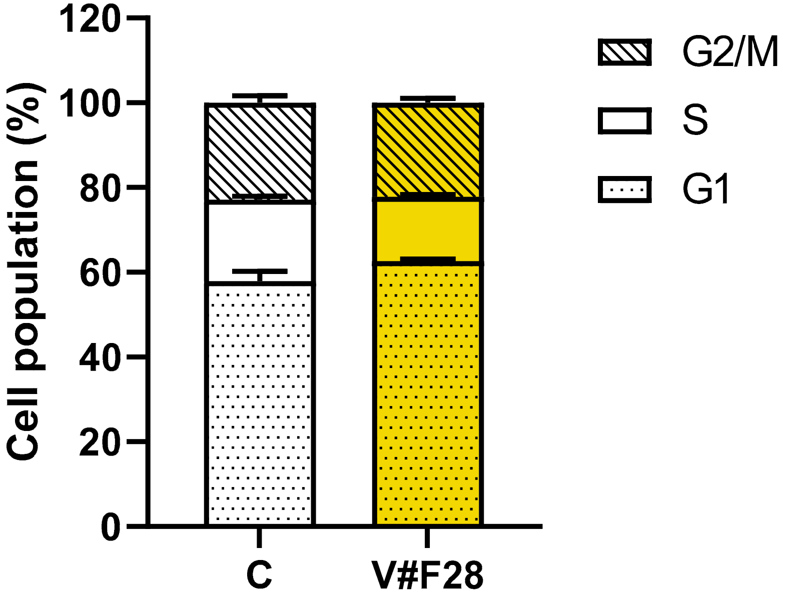

Figure S2: Analysis of the effect of V#F28 aptamer in cell cycle at 48 hours. MCF7 cells were transfected with 50 nM aptamers. After 48 hours cells were stained with PI and subjected to flow cytometry analysis. The percentage of cells gated in each phase is indicated

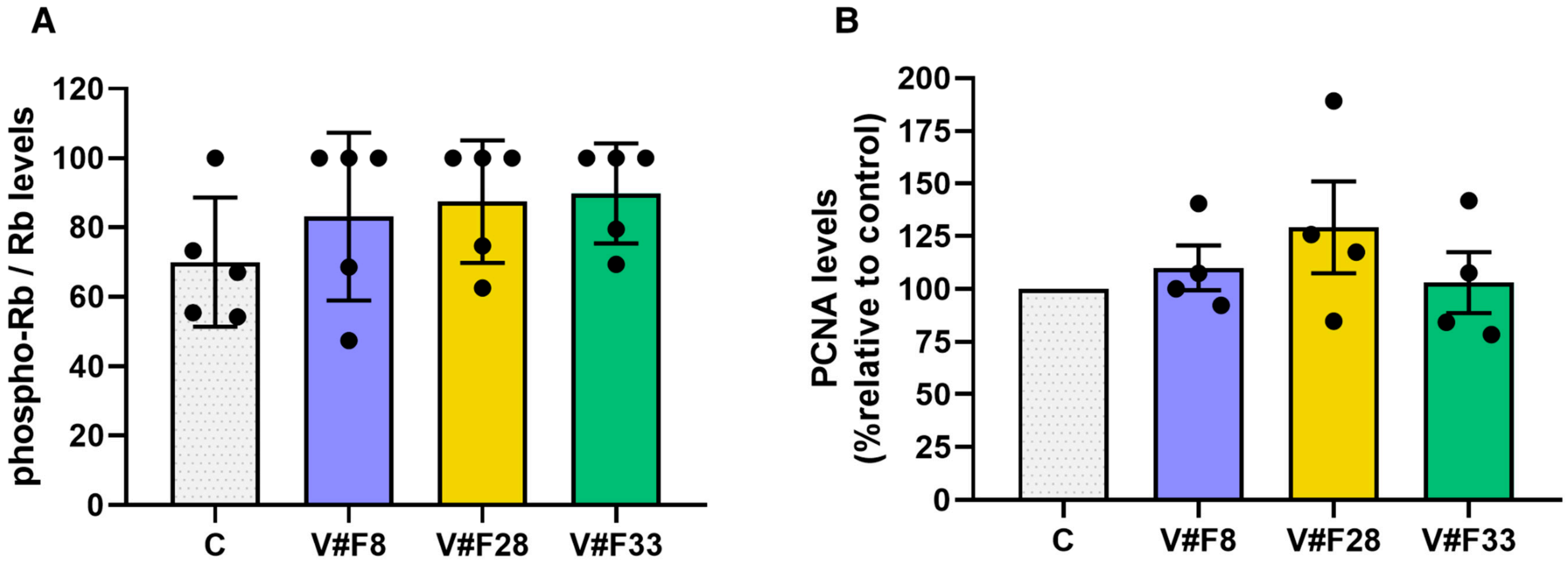

Figure S3: Levels of the phosphorylated Rb (A) and PCNA (B) after the treatment with the aptamers. A) The levels of phospho-Rb were calculated as the ratio between the levels of the bands that migrate slowly (pp band in Figure 5B) and the sum of the two bands, expressed as percentages. B) Western blot analysis of the cell lysates of Figure 5 using PCNA antibody. Actin was used as a control for the homogeneity of loading. The quantitation of the bands was normalized with respect to actin and expressed as the percentage relative to the value in control cells. The values represent the mean ± S.E.M. of 4-5 different experiments.
